# Children

**Published:** 1884-11

**Authors:** 


### CHILDREN.

THE appalling fact is revealed by mortuary statistics, that of all the children born more than one-half die before they reach the age of five years. In London alone there are 40,000 such deaths annually. Such frightful mortality is not from lack of medicine, but principally because parents possess too little knowledge as to the proper care of children. "If from his birth," says an eminent authority, "an infant has plenty of fresh air for his lungs, plenty of exercise for his muscles, plenty of sleep for his brain, frequent sousings of fresh water for his body, and only proper nourishment for his stomach, he will survive children's diseases and will require but little medicine.

Wash the new-born babe daily with warm rain water and glycerine or castile soap. Powder with wheat flour starch. Do not wash his clothes and napkins in soda. It causes breaking out. White lead is a poison. Change clothing frequently, and have it well aired. The head should be kept cool and the clothes loose. If there is no mother's milk give milk warmed, from one cow or goat only. For 1st month suckle every hour and a half; for 2d, every two hours; for 3d, once in three hours, etc. The stomach requires rest. The best substitute for milk is to boil good bread for two hours in water, and add a very little lump sugar. Gradually lower the temperature of the room. A nursing mother should avoid rich pastry, gravies, pepper, high seasoned food, cabbage, greens, spirits and coffee. Never give a child soothing syrup, paregoric or laudanum. They all contain morphine or opium. Do not add peppermint or gin to their food. A child should be weaned at from nine to twelve months After that time the breast does him more harm than good. Vaccinate any time after two months.

After one month infants should be kept in a well ventilated room Thermometer not 65. After three months he should be carried into the open air daily. Use no veil or covering to the head. He should sleep a great deal. Do not fail to bathe him thoroughly every day, and gradually get the water a little cool. There are on the human body over two million three hundred thousand perspiration pores ; 1000 to the square inch on the chest, forehead, neck, arms, back of hands and feet, and 2700 to the square inch on the palms of the hands and soles of the feet. Of the food and drink taken by an average man he discharges thirty ounces a day through the pores of his skin. Children are in this respect but small sized men. This foul matter stops the pores, and is as bad as a stoppage of the bowels.

If the child's bowels become costive, or it is suffering from fever dependent upon teething or otherwise, or it is troubled with worms, wind colic or diarrhoea, we take pleasure in recommending Castoria to mothers as an excellent and very exceptional medicine. No medicine should be given to a child without the mother's full knowledge of its character. Castoria is a standard prescription of a distinguished physician—old Dr. Pitcher—still living at Hyannis, Mass. A list of its ingredients accompanies each bottle. It is purely vegetable and is not narcotic. Of this preparation physicians speak in high terms. H. A. Archer, M. D., 111 So. Oxford St,, Brooklyn, N. Y., says : "Castoria is so well adapted to children that I recommend it as superior to any prescription known to me." Alex. Robertson, M. D., 1057 Second Ave., N. Y., says : "Especially adapted to diseases of children."

**The Sheldon,** the largest and best-appointed hotel in Ocean Grove, N. J., remains open all the year. The winter guests form a pleasant community, and life there is said to be very enjoyable and comfortable in winter as well as in summer. Winter charges are of course much reduced, and the accommodations are far more ample for the guests than during the summer, when the house is crowded.

A gentleman, the other day, presented to a young lady of his acquaintance, one of those pretty and elegant little cases containing a nail polisher, scissors, cosmetics, and other implements for keeping the hands and nails in good order ; and now they do not speak. She returned his gift as an insulting suggestion to her that her nails needed cleaning. He then sent the case to another young lady, who was not so sensitive, for she kept it, and made acknowledgment by forwarding him a cake of scented soap. And now, strangely enough, his feelings are very similar to those of the first young lady.

Unselfish people are always polite, because good manners are only the absence of selfishness.

